# Antioxidant Effect of Pumpkin Flower (*Cucurbita maxima*) in Chicken Patties

**DOI:** 10.3390/foods11152258

**Published:** 2022-07-28

**Authors:** Eva María Santos, Jose A. Rodriguez, Jose M. Lorenzo, Alicia C. Mondragón, Mirian Pateiro, Evelin Gutiérrez, Thania Alexandra Ferreira

**Affiliations:** 1 Área Académica de Química, Universidad Autónoma del Estado de Hidalgo, Carr. Pachuca-Tulancingo Km. 4.5, Mineral de la Reforma 42184, Mexico; emsantos@uaeh.edu.mx (E.M.S.); josear@uaeh.edu.mx (J.A.R.); 2 Centro Tecnológico de la Carne de Galicia, Rúa Galicia n° 4, Parque Tecnológico de Galicia, San Cibrao das Viñas, 32900 Ourense, Spain; jmlorenzo@ceteca.net (J.M.L.); mirianpateiro@ceteca.net (M.P.); 3 Área de Tecnología de los Alimentos, Facultad de Ciencias de Ourense, Universidad de Vigo, 32004 Ourense, Spain; 4 Laboratorio de Higiene Inspeccion y Control de Alimentos, Departamento de Quimica Analitica Nutricion y Bromatologia, Universidad de Santiago de Compostela, 27002 Lugo, Spain; alicia.mondragon@usc.es; 5 Departamento de Ingenieria Mecatronica, Universidad Politécnica de Pachuca, Ex. Hacienda Sta. Barbara, Zempoala 43830, Mexico; evgutierrez@upp.edu.mx

**Keywords:** pumpkin flowers, foam-mat drying, antioxidant capacity, chicken patties, edible flowers, sensory analysis

## Abstract

In this work, the antioxidant effect of pumpkin flower powder was evaluated in chicken patties. For this purpose, three drying methods were proposed to obtain the pumpkin flower powder and preserve its properties (antioxidants, color, odor): foam-mat drying, freeze drying, and oven drying. The drying process of the powder plays an important role in the conservation of bioactive compounds. The foam-mat drying method would allow the preservation of these compounds after cooking and after cold storage due to encapsulation like mechanism of the added proteins. Thus, these powders were selected as the most adequate vehicle to incorporate in the formulation, since patties with these additives presented the better antioxidant scores for DPPH, ABTS, and FRAP even after 7 days of storage. In addition, total polyphenolic content and the presence or thiobarbituric acid reactive substances (TBARS) were better scored in samples with the pumpkin flowers. The incorporation of the pumpkin flower additives in the patty formulation improved sensorial attributes of the chicken patties and consumers acceptance after cold storage.

## 1. Introduction

Lipid oxidation is considered one of the major deterioration processes affecting nutritional content and sensory attributes in food [1] and it is responsible for the quality of meat products [2]. The products from oxidation processes can change the smell, flavor, and other sensorial attributes of meat. In this regard, lipid stability in meat products frequently rely on the addition of antioxidant compounds to the formulations [3]. These compounds exhibit biological activity and play an important role in meat preservation, therefore are widely used in the food industry [4]. The synthetic antioxidants such as tert-butylhydroquinone, butylated hydroxyanisole, and propyl gallate have been used in meat and poultry products. However, several safety concerns have been raised about their presence in foods, and their use has been limited by international organizations such as the U.S. Food and Drug Administration and the World Health Organization [1,5,6]. Thus, the research on the identification of novel antioxidants coming from natural sources has recently gained the interest of food and health professionals [1,7]. Fruits, vegetables, and plants can be considered a great source of natural antioxidants due to their high phenolic content and could provide an alternative to currently used antioxidants [6]. Some described examples of antioxidants from natural sources that have been incorporated in meat and poultry products to extend the shelf-life are coffee [5], grape seed extract, pomegranate, and cranberry [6], algae [8], mushroom flours [9], hibiscus [10], and kecombrang [11].

In recent years the study of edible flowers has demonstrated that they are a great source of functional compounds, with uses in food and folk medicine [1]. Some examples of edible flowers used as additives in chicken products to enhance antioxidant activity include lavender [12], *Moringa oleifera* [13], and roselle [14]. Related to the antioxidant properties, edible flowers have been described to possess a greater ability compared with most fruits and vegetables due to their bioactive profiles [15,16]. Pumpkin flower (*Cucurbita maxima*), commonly used in the cuisine of Mexico in traditional dishes, has been recently discovered as a source of interesting biocompounds. According to Ghosh et al. [17], these flowers are rich in minerals, polyunsaturated fatty acids, and antioxidants. However, no information is available about the possible applications of pumpkin flower powder as an additive in meat products.

In general, the preservation of nutrients and bioactive compounds in edible flowers requires proper processing techniques. The common procedure is drying at elevated temperatures, which efficiently inhibits spoilage caused by microbes and enzymes [16]. However, the temperatures used in the drying process can be a critical variable since nutrients and bioactive compounds could be affected due to thermal degradation. Novel drying methods such as foam-mat drying and freeze drying have been applied to minimize the sample exposure to elevated temperatures and are considered a better option to preserve bioactive components [16,17]. Foam-mat drying is an economical alternative, suitable for preserving heat-sensitive components in food. In this technique, food is converted into stable foams using foaming agents and stabilizers, and then dried by application of hot air [18,19,20].

In this work, three drying methods, foam-mat drying, freeze-drying, and oven drying, were evaluated to preserve pumpkin flower antioxidant attributes and its potential use as antioxidants in chicken patties.

## 2. Materials and Methods

### 2.1. Reagents and Solutions

All the solutions employed were prepared with deionized water (Milli-Q Merck, Millipore Darmstadt, Hesse, Germany) with a resistivity of 18.2 MΩ cm or greater. All chemicals used were analytical grade and used without further purification. Reagents employed for the pumpkin flower foam-mat elaboration; maltodextrin, hydroxyethyl cellulose, and egg albumin (EA) were purchased from Food Technologies Trading (Estado de Mexico, Mexico), Tween-80 was purchased from J.T. Baker (Phillipsburg, NJ, USA).

Reagents employed for the antioxidant activity assays; 2,2-Diphenyl-1-picrylhydrazyl (DPPH), 6-hydroxy-2,5,7,8-tetramethylchroman-2-carboxylic acid (Trolox), potassium persulfate, 2,4,6-tris-2-piridil-s-triazine (TPTZ), hydrochloric acid, acetic acid, Folin–Ciocalteu reagent, gallic acid, thiobarbituric acid (TBA), 1,1,3,3-tetraethoxypropane (TEP), and methanol (MeOH) were purchased from Sigma Aldrich (St. Louis, MO, USA). ABTS was purchased from Roche Diagnostics (Indianapolis, IN, USA). Trichloroacetic acid (TCA) was purchased from Meyer (Estado de Mexico, Mexico). Iron (II) sulfate, sodium carbonate, and sodium acetate were obtained from J.T. Baker (Phillipsburg, NJ, USA). Ferric (III) chloride was purchased from Merck (Darmstadt, Hesse, Germany).

### 2.2. Instrumentation

Spectrophotometric measurements were performed using a UV/Vis spectrometer Perkin Elmer Lambda 40 (Waltham, MA, USA) using the Perkin Elmer UV WinLab software. A pH/ion analyzer Oakton pH510 series (Vernon Hill, IL, USA) was used to adjust the pH of the buffer solutions to 0.01 pH units. Color measurements were performed using a portable colorimeter (Konica Minolta CM-600d, Osaka, Japan) under D65 illuminant and 10° observer angle.

### 2.3. Preparation of Pumpkin Flower Powder

Fresh male pumpkin flowers (*Cucurbita maxima*) were purchased in a local market in Pachuca (Hidalgo, Mexico). These edible flowers are usually harvested when they are still a tender bud. Male flowers only contain a stamen with pollen inside, which in this case was removed along with the stem. The flowers were rinsed with distilled water and drained before use. Three different drying methods were studied for the obtention of pumpkin flower additive, in order to evaluate the persistence of the antioxidant characteristics in the pumpkin flowers:

1. Foam-mat drying
  In this method, 17.5 g of pumpkin flowers were mixed with 100 mL of water and the mixture was blended in a food blender. For the foam formulation, 100.0 g of the extract were mixed with 15.0 g of albumin, 10.0 g of maltodextrin, 2.0 g of hydroxyethyl cellulose, and 2.0 g of Tween-80 [11,17,18,19,20]. The mixture was whipped for 15 min until frothing using a hand-mixer. The sample was dried in an oven at 60 °C for 4 h and then the foam mat was ground with a grinder to obtain a powder. It is worth mentioning that two extracts were tested, one of the extracts was prepared from fresh flowers (FF) and the other from previously frozen pumpkin flowers at −18 °C (CF).
2. Freeze drying
  Approximately 100 g of pumpkin flowers were weighed. The petals, pistil, and pollen were considered in the elaboration of the additive. Freeze drying was performed in a FreeZone 6 Liter Benchtop Freeze Dry System (Labconco, MO, USA), and the obtained flowers were ground in the same way as foam-mat drying to obtain a powder (LF).
3. Oven drying
  Approximately 100 g of pumpkin flowers (considering petals, pistil, and pollen) were weighed and placed in a refractory container. The drying process was carried out in an oven at 65 °C for 12 h. The flowers were then ground to obtain a powder (OF).

### 2.4. Chicken Patties Elaboration

Five formulations with the addition of the pumpkin flower additives were designed for the chicken patties. The batches were composed of chicken breast meat, NaCl, binding protein, and pumpkin flower. The chicken meat was acquired from a local provider and ground in a meat mincer (Torrey, Mexico). FF and CF powders were obtained by the foam-mat drying method using fresh pumpkin flowers and frozen flowers, respectively, the foaming agent was egg albumin, which also enriched the protein content of the powder. LF was obtained by freeze drying and OF by oven drying. In this sense, the control batch contained 1.2% NaCl and 1.5% soy protein, FF: 1.2% NaCl and 1.5% FF, CF: 1.2% NaCl and 1.5% CF, LF: 1.2% NaCl, 1.45% soy protein, and 0.05% LF, OF: 1.2% NaCl 1.45% soy protein and 0.05% OF.

FF and CF batches included 1.5% of the flower powder, since this additive contains egg albumin (EA) as a protein source, LF and OF additives consisted only of the dried pumpkin flower. In this case, the protein source added was soy protein (1.45%) [9]. Next, 80 g portions of chicken meat were molded in a hamburger maker with a diameter of 78 and 10 mm height. The cooking of the chicken patties was carried out in an industrial air oven (Rational, Landsberg am Lech, Germany) at 79 ± 3 °C to reach a core temperature of 69 °C. Antioxidant assays and color evaluation were performed on days 0 and 7, before and after cooking.

### 2.5. Sample Analyses

The evaluation of the antioxidant profile of the pumpkin flower additives and the chicken patty samples was carried out by different methodologies. It has been described that DPPH, ABTS, and FRAP methodologies provide complementary information about antioxidant properties [21].

Chicken patty formulations were elaborated, and samples were taken at two different times to evaluate the influence of the pumpkin flowers drying method on the antioxidant activity during storage: Fresh raw formulation (day 0), cooked patties (day 0), raw formulation after 7 days of storage, and cooked patties after 7 days of storage. Methanolic extracts from samples were used for the evaluation [4,17]. To obtain the extracts, 2.0 g of chicken patty were weighed in polypropylene tubes and added with 5.0 ± 0.1 mL of MeOH. The mixtures were left under ultrasound for 10 min, centrifuged at 2200 rpm, and filtered with paper (Whatman 41). All analyses were performed in triplicate.

DPPH method was performed according to Rivero–Perez et al. [22]. The decrease of absorbance is monitored spectrophotometrically at 515 nm when antioxidant species are added to DPPH radical. The results obtained for the antioxidant capacity were expressed as the inhibition percentage [17,23] according to Equation 1.

(1) $$\%\mathrm{Inhibition}=\frac{{\mathrm{Abs}}_{\mathrm{control}}-{\mathrm{Abs}}_{\mathrm{sample}}}{{\mathrm{Abs}}_{\mathrm{control}}}\times \;100$$

The ABTS method was applied as described by Rivero–Perez [22] based on the decolorization that occurs when an antioxidant reacts with ABTS radical cation (ABTS^+^•). A decrease in absorbance was measured at 734 nm. The results were expressed as the inhibition percentage. The FRAP method was performed according to Benzie et al. [23]. The concentration of antioxidant compounds is related to the increase in the absorbance at 593 nm. The results were expressed as mmol FeSO_4_•100 kg^−1^ chicken patty considering dry matter.

In addition to the antioxidant capacity, total polyphenol content was determined using the Folin–Ciocalteu reagent (FCR) [22]. Gallic acid was used as a standard and the results were expressed as mg_gallic acid_ g^−1^ chicken patty.

Lipid oxidation was evaluated following the development of thiobarbituric acid reactive substances (TBARS) according to the procedure described by Vyncke [24] with some modifications. Once the aqueous extract was obtained, it was cleaned using a C_18_ SPE cartridge (Supelco, Discovey DSC-18) to retain the phenolic compounds [25]. Subsequently, the filtrate was analyzed following the TBARS protocol. Chicken meat contains fatty acids that are susceptible to oxidative degradation. Lipid oxidation results in aldehydes as one of the majority products, which can affect the sensorial attributes of meat and its nutritional content [3]. TBARS values were expressed as mg _MDA_ kg^−1^ chicken patty.

### 2.6. Moisture and Color Measurements

Moisture content in chicken patties was measured according to the Association of Analytical Communities (A.O.A.C.) moisture determination method (23.003:2003), by weighing 2.000 ± 0.001 g of the sample. These samples were dried in oven at 105 °C up to constant weight. Each sample was measured in triplicate.

CIEL*a*b* parameters L*, a*, b* (L: color lightness; a: redness; b: yellowness) were determined directly on four different points on the surface of the sample. Average color values were expressed.

### 2.7. Sensory Evaluation

Sensory evaluation of the chicken patties was performed using a 5-point hedonic test. The attribute test was conducted by fifteen trained panelists [8,9]. Samples were identified by three-digit numbers and presented in random order to the panelists. Hedonic scores ranged from 1 to 5 representing from very unpleasant (1) to excellent (5). The test included the evaluation of color, odor, texture, taste, and overall acceptability [8]. For this evaluation, chicken patties were cooked at 69 °C internal temperature using fresh formulations and formulations after seven days of storage in refrigeration (4 °C). The samples were presented to each panelist in plastic dishes, offering water to clean the palate from residual flavors.

### 2.8. Statistical Analyses

Results of the sample analyses were presented as the mean and standard deviation of three replicates (*n* = 3). Statistical analysis of the data obtained was performed using Minitab 17 software. Mean values were compared using the one-way ANOVA and Tukey multiple range tests were used to estimate the level of significance among chicken patties [3]. Principal component analysis (PCA) was performed to outline differences and groupings among samples. Methodology and storage time (raw and cooked patties on day 0 and day 7) were used as variables.

## 3. Results and Discussion

### 3.1. Pumpkin Flower Additives

**Figure 1:**
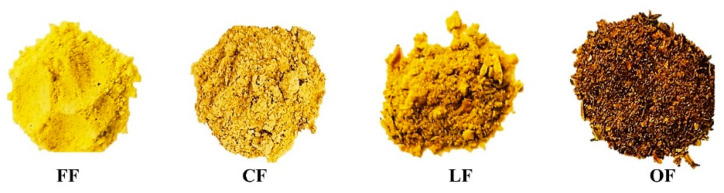
Pumpkin flower additives FF: fresh pumpkin flower foam, CF: frozen pumpkin flower foam, LF: freeze-dried pumpkin flower, OF: oven-dried pumpkin flower.

Four formulations were developed to be evaluated as chicken patties additives to provide color and increase antioxidant activity in the final product (Figure 1). At first sight color differences were appreciated and also revealed in the CIEL*a*b* parameters (data not shown). OF powder presented the darkest color because of the drying process without any protector as it happens in freeze drying and foam-mat dying process [19,20].

### 3.2. Antioxidant Capacity

There are no previous data available for the antioxidant capacity of pumpkin flower additives and their application in meat products [17]. Studies were performed using three methodologies, DPPH, ABTS, and FRAP. The results obtained are shown in Table S1 considering dry weight of the samples. The results observed in each antioxidant methodology depend on their mechanism and sensibility, DPPH has been described for the determination of lipophilic antioxidants, while FRAP for hydrophilic antioxidants and ABTS for both types [26].

**Figure 2:**
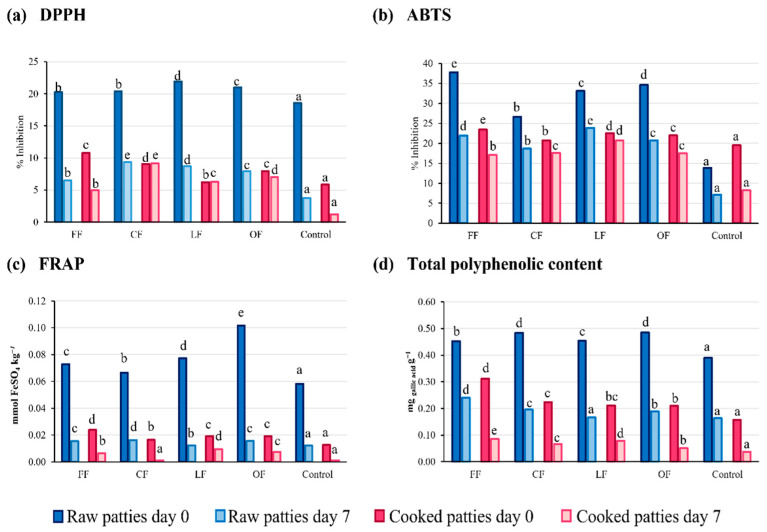
Effect of the addition of pumpkin flower on the antioxidant activity measured in chicken patties with (**a**) DPPH method, (**b**) ABTS method, (**c**) FRAP method, (**d**) total polyphenolic content method. Different letters represent statistically significant differences (*p* < 0.05) between formulations.

As shown in Figure 2, in fresh chicken patties a significant improvement in the antioxidant properties was observed with the incorporation of the pumpkin flower additives (*p* < 0.05). Thermal treatment of the patties and microbial growth accelerated degradation and favored oxidation phenomena that affect naturally occurring reducing compounds. Control samples showed the presence of antioxidant compounds to a lesser extent, according to Arshad et al. the incorporation of antioxidant species in the feeding reflects in the antioxidative parameters in the meat product [27]. After cooking, a significant (*p* < 0.05) decrease in antioxidant activity was observed. Nevertheless, the formulations containing the additives continued presenting a higher score compared to the control samples. After cold storage, this trend remained constant. The FF, CF, and LF formulations exhibited the higher antioxidant activity.

The content of phenolic compounds in the patties was also increased by the addition of pumpkin flower additives. It is possible to observe the presence of polyphenolic compounds in chicken meat, which agrees with the information previously described by Madane et al. [13]. A reason for looking at the presence of polyphenols in chicken meat may be that they can be used as feed to improve gut health in broilers due to their health benefits and antioxidant potential [28]. After the addition of the pumpkin flower additives, TPC significantly increased. The highest polyphenolic content after cooking was found in the FF formulation (0.312 ± 6.12 × 10^−4^ mg_GAE_ kg^−1^). The lowest concentration of polyphenolic compounds was found in the control group (0.157 ± 7.84 × 10^−4^ mg_GAE_ kg^−1^).

**Figure 3:**
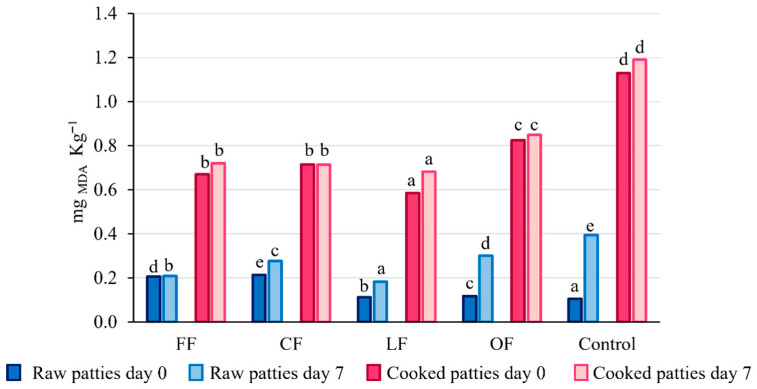
Effect of the addition of pumpkin flower on the content of thiobarbituric reactive substances (TBARS assay). Different letters represent statistically significant differences (*p* < 0.05) between formulations.

Lipid stability is a vital parameter that determines the quality of meat and meat products because of its effect on protein oxidation and discoloration of the meat. In this sense, it was assessed with the thiobarbituric reactive substances method (TBARS) (Figure 3). The addition of the pumpkin flower additives in the formulation prevented the oxidation process of lipids during storage and cooking compared with the control sample. During cooking, the oxidation reactions are accelerated, but an inhibitory effect on the meat was clearly seen, especially when using FF, CF, and LF additives. In addition, during the storage lipid oxidation in the control, the evaluated samples increased significantly (*p* < 0.05). Raw and cooked patties containing the pumpkin flower presented lower TBARS values compared to the control. This may be due to the effect of polyphenols in the additives [17]. Generally, the TBARS values in all patties were significantly increased with the cooking process and with cool storage. These findings are in agreement with previous results using cantaloupe and other fruits or plants as additives in chicken patties regarding the inhibition of lipid oxidation during storage [13,29,30]. Madane et al. evaluated the incorporation of *Moringa oleifera* flower in chicken nuggets and its effect on TBARS content during storage. They described MDA concentrations in raw samples from 0.36 ± 0.01 to 0.84 ± 0.02 mg _MDA_ kg^−1^, these values are higher than the ones obtained in this study with the pumpkin flower [13]. Hwang et al. described the addition of *Artemisia princeps* in chicken patties. The evaluation of raw patties presented TBARS values below 0.4 mg _MDA_ kg^−1^ after 7 days of storage [30], similar results were obtained in this work. Generally, TBARS values of 2.0 mg _MDA_ kg^−1^ are considered acceptable for human consumption [31]. In this study, all the formulations have acceptable levels during the evaluation period. (See also Table S1. Results obtained from the evaluation of antioxidant capacity in chicken patties).

According to the results obtained in the analysis of antioxidants, the drying process of the powder plays an important role in the conservation of bioactive compounds. Oven drying involves the use of high temperatures for long periods of time without the presence of any compound that would protect the bioactive compounds present in the flower and allow their adequate conservation. Thus, despite providing antioxidant compounds to chicken patties, this was to a lesser extent compared to the rest of the drying methods evaluated. Freeze drying is a good option since it allows to improve the inhibition of lipid oxidation processes in chicken patties, but it is not affordable due to the high cost, and it is time-consuming compared to foams [18,19,20].

The foam-mat drying method for pumpkin flower additives offers advantages since this method would allow the preservation of bioactive compounds after cooking and after cold storage compared with the conventional drying method due to the encapsulation-like mechanisms of the added proteins [18,19]. On the other hand, it is an economical, simple, and fast alternative to obtain pumpkin flower powder and adds protein to the additives by incorporating egg albumin as a foaming agent.

Formulations containing FF and CF additives presented the highest values for antioxidant determination and polyphenolic content, as well as freeze-dried powder LF. Despite presenting a decrease in antioxidant activity after the cooking process and after 7 days of storage, this behavior positively influenced the preservation of the chicken patty by minimizing its oxidative degradation. This was reflected in the lipid oxidation and in the organoleptic properties.

**Figure 4:**
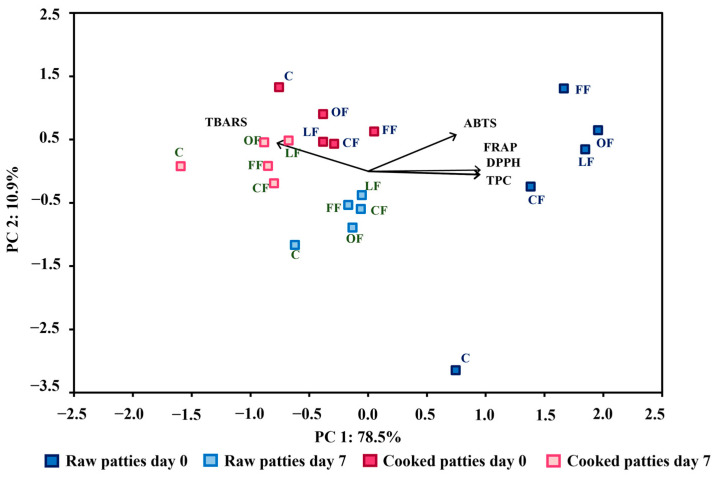
PCA analysis for the antioxidant activity and lipid oxidation in raw and cooked chicken patties.

The principal component analysis (PCA) of the factors and variables analyzed in chicken patties is shown in Figure 4. The principal components 1 and 2 accounted for 78.5% and 10.9% of variance, respectively. Patties were clearly distinguished between raw and cooked. Fresh raw patties presented the highest antioxidant activity. When samples were cooked, antioxidant concentrations decreased, and lipid oxidation increased. The cooked samples with 7 days of storage presented the highest values of TBARS. In addition, control samples exhibited higher TBARS comparing the other treatments. According to the results, pumpkin flower additives reacted with oxidative compounds in order to reduce the oxidation of lipids in the chicken patties in comparison with the control.

### 3.3. Moisture and Color Measurements

Moisture content was also determined in the chicken patties and the results are shown in Table 1. The incorporation of foam-mat additives CF and FF in chicken patties did not significantly change the moisture content (*p* > 0.05). On the contrary, the addition of LF and OF decreased the moisture content of the patties compared with the control. Serdaroğlu et al. [32] evaluated the addition of dried pumpkin pulp and seeds in beef patties also obtaining a decrease in the moisture content. Moisture retention in chicken patties after cooking was observed ranked between 75.67% (Control) and 82.01% (OF) but LF and OF samples again presented significantly lower moisture values (*p* < 0.05). Incorporation of foam-mat dried pumpkin additives favored the moisture retention compared with the control due the addition of fiber and protein as they form gel networks with meat proteins which helps trap water within the matrix [9,32,33,34].

**Figure 5:**
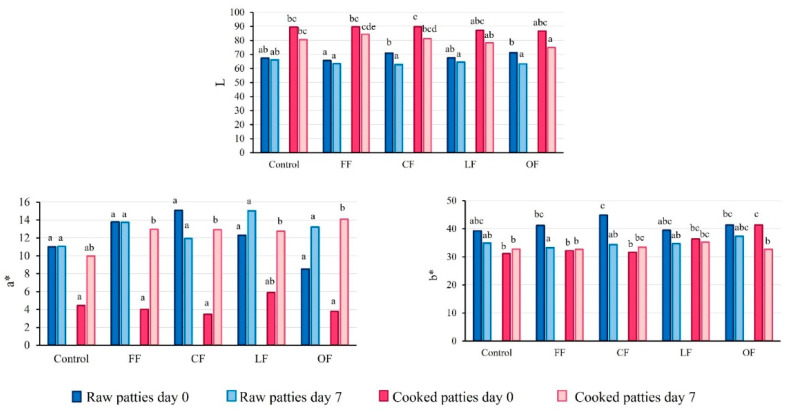
L* Lightness, a* value (redness), and b* value (yellowness) for raw and cooked chicken patties measured at day 0 and day 7. Different letters represent statistically significant differences (*p* < 0.05) between formulations.

Color is one of the most important parameters for determining consumer acceptance of food products. In this work, the effect of the presence of pumpkin flower additives in the chicken patties was evaluated and all the chicken batches were formulated under the same experimental conditions. Color properties L*, a*, b* of raw and cooked patties are shown in Figure 5.

As can be observed, it was possible to observe an increase in the lightness (L* value) of the samples after cooking, and a decrease in this attribute after 7 days of conservation, and these differences are statistically significant according to a Tukey test (*p* > 0.05).

The a* values decreased drastically after cooking due to oxidation phenomena of myoglobin into metmyoglobin and myohemochromogen/myohemichromogen. However, this effect was not noticeable after 7 days of storage. LF and OF have higher a* values after cooking. The b* values also decreased during storage, but variations were less pronounced after cooking.

**Figure 6:**
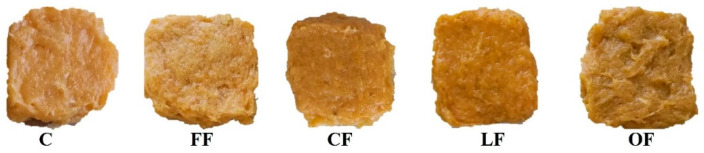
Differences in color produced by the addition of the pumpkin powders in the raw patties compared with the control.

Despite the differences observed in the data, the main color changes observed in this study were produced by the cooking process and by degradation effects in the product after storage, more than the effect of the addition of pumpkin flower additive as Figure 6 shows and sensorial analysis revealed.

### 3.4. Sensory Analysis

**Figure 7:**
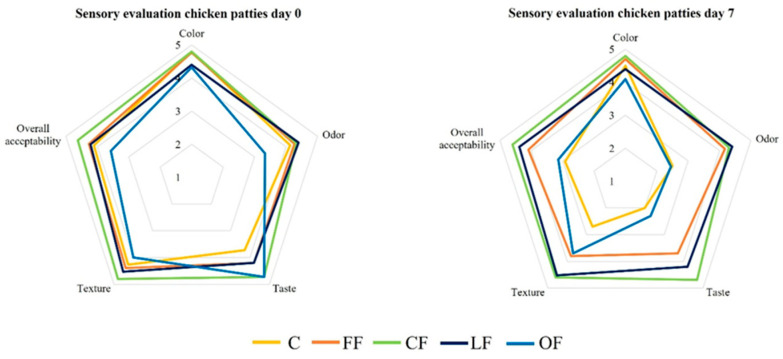
Five-point hedonic test results for the evaluation of chicken patties added with pumpkin flower additives.

Sensory evaluation of the chicken patties was carried out on the cooked patties on day 0 and day 7 of storage. The results are shown in Figure 7. On the first day, hardly any differences were found. The panelists gave the highest score to CF formulation with an overall acceptability of 4.62, while the OF formulation was scored 3.57 for overall acceptability (*p* < 0.05). In general, in comparison with the control, color, and texture were not affected (*p* > 0.05) by the presence of the pumpkin flower. For odor, FF, CF, and LF formulations presented the most attractive aroma to panelists (>4.27) while the OF formulation received the lowest score (3.33) although it was still acceptable. The aroma of pumpkin flowers was slightly perceived. In comparison with the addition of other additives to chicken products, and their effect on sensory evaluation, Feridoni et al. evaluated the addition of *Hibiscus sabdariffa* L. in chicken nuggets, and they observed via the color and overall acceptance that sensory scores were significantly reduced by adding preservatives [14], while the odor and taste were not affected.

After 7 days of storage (4 °C) no changes in color were appreciated between treatments by the panelist compared to the instrumental measurements, while odor, taste, and texture changes were noticeably appreciated. Lipid oxidation during storage causes rancidity and the consequent bad odor and taste in the chicken patties. The control and OF samples presented the lowest scores in odor and taste (*p* < 0.05). Related to odor, the panelists perceived a slightly unpleasant aroma, described as a decomposition smell, in the control and OF formulations and to a lesser extent, FF, while no unpleasant odors were perceived in CF and LF patties. Although microbiological analysis was not done, maybe a certain antimicrobial effect could also be expected in the additives to improve the preservation of the product as has been observed with other flowers like *Rosselle* [10]. In the evaluation of taste, a similar pattern was observed. CF and LF did not show significant differences in comparison with fresh patties but OF, and control patties were scored as un-pleasant for the panelists. According to the texture evaluation, control patties had the lowest scores as panelists described them with a chewy texture. The odor and taste scores negatively affected the overall acceptability but even the samples worst evaluated presented values near acceptable levels. According to the results, the addition of pumpkin flower prepared by a foam mat and freeze dried hardly affected the patties on the elaboration day but contributed to better scores after one week of storage. Concerning overall acceptability, the patties followed the same pattern as described by Madane et al., with the incorporation of *Moringa oleifera*, and Feridoni et al., with the addition of *Hibiscus sabdariffa*. The control sample received the lowest overall acceptability score after storage and was found to develop a rancid odor [13,14].

## 4. Conclusions

To the best of our knowledge, this is the first work in using pumpkin flower (*Cucurbita maxima*) as an antioxidant additive in meat, and in particular chicken patties formulations. According to the DPPH, ABTS, and FRAP, total polyphenol content results, it seems that the antioxidant properties of pumpkin flower are effective to reduce the oxidation processes occurring during the cooking and storage of patties, improving the sensorial scores after cooking. Even though the three methods evaluated exhibited an increased antioxidant capacity, the foam-mat and freeze-drying processes resulted in better antioxidant and sensorial effects, while oven-drying was not so effective. At this point, the certain antimicrobial activity could be inferred considering control and OF samples presented higher spoilage, but the antimicrobial activity of the additives should be studied in the future in order to confer both aspects. Considering the results and the fact that foam-mat drying is a simple, efficient, and cheaper method to obtain the additives, foam-mat drying of fresh pumpkin flowers should be the best option to be incorporated in chicken patties.

## Figures and Tables

**Table 1:** Moisture content in chicken patties.

	Control	FF	CF	LF	OF
Moisture in raw patties (*n* = 3)	34.08 ± 0.90 ^a^	34.41 ± 0.41 ^a^	34.93 ± 0.69 ^a^	29.20 ± 0.94 ^b^	29.20 ± 0.14 ^b^
Moisture in cooked patties (*n* = 3)	25.79 ± 0.40 ^a^	26.16 ± 0.67 ^a^	26.92 ± 0.35 ^a^	23.06 ± 0.38 ^b^	23.97 ± 0.80 ^b^

Different letters represent statistically significant differences (*p* < 0.05) between formulations.

## Data Availability

Data is contained within the article or supplementary material.

## Supplementary Materials

The following supporting information can be downloaded at: https://www.mdpi.com/article/10.3390/foods11152258/s1, Table S1: Results obtained from the evaluation of antioxidant capacity in chicken patties.
