# Supplementary material for: Antioxidant Effect of Pumpkin Flower (Cucurbita maxima) in Chicken Patties
Source: Foods. 2022 Jul 28;11(15):2258. doi: 10.3390/foods11152258 (PMC9368278; doi:10.3390/foods11152258)
Supplement: Supplementary file 1 [file foods-11-02258-s001.zip › foods-1822495-supplementary.pdf]

Table S1. Results obtained from the evaluation of antioxidant capacity in chicken patties

**Day 0**	Raw patties					Cooked patties				
	C	FF	CF	LF	OF	C	FF	CF	LF	OF
DPPH (% inhibition)	18.610 ± 0.070^{d}	20.290 ± 0.070^{c}	20.410 ± 0.070^{c}	21.920 ± 0.150^{a}	21.000 ± 0.150^{b}	5.860 ± 0.070^{e}	10.790 ± 0.070^{a}	9.060 ± 0.150^{b}	6.240 ± 0.150^{d}	7.920 ± 0.070^{c}
ABTS (% inhibition)	7.140 ± 0.160^{e}	37.830 ± 0.140^{a}	26.670 ± 0.160^{d}	33.100 ± 0.140^{c}	34.590 ± 0.080^{b}	19.520 ± 0.080^{e}	23.460 ± 0.160^{a}	20.720 ± 0.140^{d}	22.530 ± 0.240^{b}	21.970 ± 0.140^{c}
FRAP (mmol $FeSO_4$ kg^{-1})	0.583 ± 0.002^{e}	0.729 ± 0.006^{c}	0.665 ± 0.010^{d}	0.774 ± 0.006^{b}	1.017 ± 0.006^{a}	0.127 ± 0.002^{d}	0.239 ± 0.002^{a}	0.160 ± 0.002^{c}	0.192 ± 0.002^{b}	0.193 ± 0.004^{b}
Polyphenolic content ($mg_{gallic\ acid}$ g^{-1})	0.433 ± 0.001^{d}	0.450 ± 0.001^{c}	0.483 ± 0.001^{a}	0.545 ± 0.001^{b}	0.485 ± 0.001^{a}	0.157 ± 0.001^{d}	0.312 ± 0.001^{a}	0.224 ± 0.001^{b}	0.213 ± 0.001^{bc}	0.210 ± 0.002^{c}
TBARS (mg_{MDA} kg^{-1})	0.107 ± 0.002^{e}	0.206 ± 0.002^{b}	0.214 ± 0.002^{a}	0.112 ± 0.002^{d}	0.118 ± 0.002^{c}	1.130 ± 0.004^{a}	0.670 ± 0.002^{c}	0.715 ± 0.003^{c}	0.588 ± 0.003^{d}	0.824 ± 0.004^{b}

**Day 7**	**Raw patties**					**Cooked patties**				
	**C**	**FF**	**CF**	**LF**	**OF**	**C**	**FF**	**CF**	**LF**	**OF**
DPPH (% inhibition)	3.750 ± 0.070^{e}	6.520 ± 0.070^{d}	9.370 ± 0.130^{a}	8.700 ± 0.070^{b}	7.910 ± 0.070^{c}	1.220 ± 0.070^{e}	4.970 ± 0.070^{d}	9.190 ± 0.070^{a}	6.280 ± 0.070^{c}	7.000 ± 0.150^{b}
ABTS (% inhibition)	13.88 ± 0.080^{e}	21.930 ± 0.080^{b}	18.700 ± 0.080^{d}	23.880 ± 0.160^{a}	20.700 ± 0.160^{c}	8.230 ± 0.080^{d}	17.050 ± 0.150^{c}	15.580 ± 0.080^{b}	20.720 ± 0.130^{a}	17.540 ± 0.130^{b}
FRAP (mmol $FeSO_4$ kg^{-1})	0.122 ± 0.002^{d}	0.155 ± 0.002^{b}	0.163 ± 0.002^{a}	0.124 ± 0.001^{c}	0.157 ± 0.002^{b}	0.011 ± 0.001^{d}	0.066 ± 0.002^{c}	0.011 ± 0.001^{d}	0.096 ± 0.002^{a}	0.075 ± 0.003^{b}
Polyphenolic content ($mg_{gallic\ acid}$ g^{-1})	0.163 ± 0.001^{d}	0.240 ± 0.001^{a}	0.196 ± 0.001^{b}	0.166 ± 0.001^{d}	0.189 ± 0.001^{c}	0.038 ± 0.001^{e}	0.086 ± 0.001^{a}	0.066 ± 0.001^{c}	0.078 ± 0.001^{b}	0.051 ± 0.001^{d}
TBARS (mg_{MDA} kg^{-1})	0.396 ± 0.002^{a}	0.209 ± 0.004^{d}	0.276 ± 0.002^{c}	0.183 ± 0.002^{e}	0.300 ± 0.004^{b}	1.190 ± 0.002^{a}	0.720 ± 0.002^{c}	0.714 ± 0.003^{c}	0.682 ± 0.002^{d}	0.850 ± 0.004^{b}

C: Control, FF: foam-mat dried fresh pumpkin flower; CF: foam-mat dried frozen pumpkin flower; LF: freeze dried pumpkin flower; OF: oven-dried pumpkin flower. Different letters represent statistically significant differences ($p < 0.05$) between formulations
